# Factors in the occurrence and restoration of hypoparathyroidism after total thyroidectomy for thyroid cancer patients with intraoperative parathyroid autotransplantation

**DOI:** 10.3389/fendo.2022.963070

**Published:** 2022-07-22

**Authors:** Dengwei Lu, Enjie Tang, Supeng Yin, Junping Zhu, Hongbiao Mo, Ziying Yi, Fan Chai, Yizeng Sun, Yao Li, Tingjie Yin, Zeyu Yang, Fan Zhang

**Affiliations:** ^1^ Breast and Thyroid Surgical Department, Chongqing General Hospital, Chongqing, China; ^2^ Graduate School of Medicinel, Chongqing Medical University, Chongqing, China; ^3^ Epidemiology Department, College of Preventive Medicine, Army Medical University (Third Military Medical University), Chongqing, China

**Keywords:** postoperative hypoparathyroidism, parathyroid hormone, thyroidectomy, parathyroid glands, thyroid cancer

## Abstract

**Introduction:**

Postoperative hypoparathyroidism (POH) is the most common and important complication for thyroid cancer patients who undergo total thyroidectomy. Intraoperative parathyroid autotransplantation has been demonstrated to be essential in maintaining functional parathyroid tissue, and it has clinical significance in identifying essential factors of serum parathyroid hormone (PTH) levels for patients with parathyroid autotransplantation. This retrospective cohort study aimed to comprehensively investigate influential factors in the occurrence and restoration of POH for patients who underwent total thyroidectomy with intraoperative parathyroid autotransplantation (TTIPA).

**Method:**

This study was conducted in a tertiary referral hospital, with a total of 525 patients who underwent TTIPA. The postoperative serum PTH levels were collected after six months, and demographic characteristics, clinical features and associated operative information were analyzed.

**Results:**

A total of 66.48% (349/525) of patients who underwent TTIPA were diagnosed with POH. Multivariate logistic regression indicated that Hashimoto’s thyroiditis (OR=1.93, 95% CI: 1.09-3.42), P=0.024), the number of transplanted parathyroid glands (OR=2.70, 95% CI: 1.91-3.83, P<0.001) and postoperative blood glucose levels (OR=1.36, 95% CI: 1.06-1.74, P=0.016) were risk factors for POH, and endoscopic surgery (OR=0.39, 95% CI: 0.22-0.68, P=0.001) was a protective factor for POH. Multivariate Cox regression indicated that PTG autotransplantation patients with same-side central lymph node dissection (CLND) (HR=0.50; 95% CI: 0.34-0.73, P<0.001) demonstrated a longer time for increases PTH, and female patients (HR=1.35, 95% CI: 1.00-1.81, P=0.047) were more prone to PTH increases. Additionally, PTG autotransplantation with same-side CLND (HR=0.56, 95% CI: 0.38-0.82, P=0.003) patients had a longer time to PTH restoration, and patients with endoscopic surgery (HR=1.54, 95% CI: 1.04-2.28, P=0.029) were more likely to recover within six months.

**Conclusion:**

High postoperative fasting blood glucose levels, a large number of transplanted PTGs, open surgery and Hashimoto’s thyroiditis are risk factors for postoperative POH in TTIPA patients. Elevated PTH levels occur earlier in female patients and patients without CLND on the transplant side. PTH returns to normal earlier in patients without CLND and endoscopic surgery on the transplant side.

## Introduction

Thyroid cancer (THCA) is one of the most prevalent endocrine neoplasms, and its incidence has been constantly increasing over recent years (1). Total thyroidectomy has been regarded as the main modality of treatment for THCA, but it carries the potential for several complications (2). Postoperative hypoparathyroidism (POH) is the most common and important complication and is associated with decreased serum parathyroid hormone (PTH) levels, hypocalcemia and hyperphosphatemia (3). The reported incidence of POH is 30%~68%, with a 6-month transit rate of 19%-38% and a lifetime rate of 0-20% (4, 5). Transient hypoparathyroidism prolongs inpatient rehabilitation and increases hospital stays and treatment costs (6). Additionally, permanent hypoparathyroidism is associated with elevated risks of infection, renal failure, psychiatric morbidities and even mortality (7). Therefore, preventing the occurrence and facilitating the restoration of POH are of vital importance, and thus, the prerequisite of identifying influential factors is imperative.

Intraoperative parathyroid gland (PTG) autotransplantation is one of the main methods used to prevent POH and reduce the rate of permanent hypoparathyroidism (8). However, studies have indicated that the occurrence rate of POH remains 30% ~ 68% for patients with intraoperative transplantation, provoking confusion about what influences the occurrence of POH in these patients (4). According to previous studies, numerous factors, such as age, sex and surgical approaches, were reported to be associated with the occurrence of POH, indicating that complex intrinsic factors are involved in the occurrence of hypoparathyroidism (9–11). As the surgical model of thyroidectomy evolves toward a more precise paradigm of intraoperative transplantation of PTG, the associated influential factors remain to be explored.

In this study, we sought to investigate influential factors and develop a predictive model for the occurrence and restoration of hypoparathyroidism in patients who underwent total thyroidectomy with intraoperative parathyroid autotransplantation (TTIPA).

## Materials and methods

### Patients

This was a retrospective case–controlled cohort study of patients undergoing TTIPA in the Department of Breast and Thyroid Surgery, Chongqing General Hospital, China, from January 2020 to December 2020. The aim of this study was to investigate influential factors for hypoparathyroidism of THCA; therefore, all patients 18-65 years of age who underwent thyroidectomy with intraoperative parathyroid autotransplantation were included. THCA was confirmed by pathology. Exclusion criteria included incomplete information, preoperative hypoparathyroidism, and severe cardiovascular or respiratory diseases. This study was approved by the Ethics Review Board of Chongqing General Hospital. Written informed consent was obtained from all participants.

### Surgical conduct for THCA at chongqing general hospital

Thyroidectomy was performed for confirmed cases of THCA with a length larger than 1 cm, surrounding invasion or distant metastases. Complete central lymph node dissection (CLND) was performed on the side of the THCA. A contralateral central neck dissection followed if positive lymph nodes were detected in the central contents by frozen-section pathological examination. Moreover, concurrent unilateral or bilateral modified lateral neck dissection was performed if lateral neck lymph node metastases were considered according to preoperative examinations. All surgeries were performed by one experienced surgeon who performs over 1000 thyroid surgeries per year.

### Parathyroid gland identification and transplantation

Parathyroid glands were routinely identified by the naked eye or ICG combined with autofluorescence if it was difficult to evaluate their location and blood supplies. Detailed methods of ICG combined with autofluorescence were described in our previous study (12). If the blood supply was deemed sufficient by the surgeon’s verdict, the suspected parathyroid would be preserved in situ. Autotransplantation was performed when inadvertent parathyroidectomies or devascularization were performed. Suspected parathyroid glands were stored in saline at 4°C for autotransplantation after pathological confirmation. The parathyroid glands were minced into less than 0.5 mm pieces and transplanted into sternocleidomastoid.

### Data collection

All data, including the general information, clinical features and surgical information, were collected retrospectively and are shown in **Table 1**. Patients received follow-up every 2-3 months. At each follow-up visit, patients were tested for serum PTH, calcium and phosphorus levels.

**Table 1:** Pathological and clinical features of 525 patients.

Variables (continuous variables)	Mean (SD)	Variables (discrete variables)	Number(%)	Variables (discrete variables)	Number(%)	
Age (year)	39.38 (10.89)	Gender		Lateral lymph node dissection		
BMI (m^2^/kg)	23.31 (3.51)	Male	90 (17.14)	Yes (Left)	35 (6.67)	
Preoperative serum PTH (pg/ml)	50.19 (19.18)	Female	435 (82.86)	Yes (Right)	43 (8.19)	
Preoperative serum calcium (mmol/L)	2.42 (0.13)	FNA		No	447 (85.14)	
Preoperative serum glucose (mmol/L)	5.34 (0.87)	Yes	315 (60.00)	Central lymph node dissection		
Duration of operation (minute)	130.16 (44.84)	No	210 (40.00)	Left	123 (28.00)	
Intraoperative blood loss (ml)	72.91 (43.84)	BRAF V600E mutation status		Right	117 (25.00)	
Serum glucose of POD1 (mmol/L)	5.83 (1.16)	BRAF V600E ^Mutation^	145 (27.62)	Bilateral	285 (47.00)	
Post-operative drainag (ml)	129.26 (140.88)	BRAF V600E ^Wild type^	18 (3.43)	PTG autotransplantation with same side CLND		
Serum PTH of POD1 (pg/ml)	12.21 (15.02)	N/A	362 (68.95)	Yes	461 (87.81)	
Serum PTH of POD2 (pg/ml)	4.23 (4.73)	Hashimoto thyroiditis		No	64 (12.19)	
Serum PTH of POD3 (pg/ml)	3.27 (4.25)	Yes	125 (23.81)	Number of PTG autotransplantation		
Serum PTH of POD4 (pg/ml)	3.43 (4.32)	No	400 (76.19)	1	136 (25.90)	
Serum PTH within POD4 to POD16 (pg/ml)	19.09 (16.43)	Method of thyroidectomy		2	224 (42.67)	
Serum PTH within POD16 to POD60 (pg/ml)	22.29 (13.78)	Open	445 (84.76)	3	155 (29.52)	
Serum PTH within POD60 to POD180 (pg/ml)	23.09 (8.21)	Endoscopic	80 (15.24)	4	10 (1.91)	
Serum calcium of POD1 (mmol/L)	2.22 (1.59)	Lymph node metastisis, pathology		Post-operative hypoparathyroidism		
Serum calcium of POD2 (mmol/L)	2.07 (0.43)	No	251 (47.81)	No	176 (33.52)	
Serum calcium of POD3 (mmol/L)	2.00 (0.21)	Yes	274 (52.19)	Yes	349 (66.48)	
Serum calcium of POD4 (mmol/L)	2.06 (0.29)					
Average calcium intake (g/day)	0.84 (1.12)					
Average hospitalization time (day)	6.36 (1.62)					

BMI, Body Mass Index; PTH, Parathyroid hormone; POD number, Post-operative day number; FNA, Fine needle aspiration; PTG, Parathyroid gland; CLND, Central lymph node dissection.

### Definition

Hypoparathyroidism was defined as a serum PTH level <12 pg/ml (normal range: 12–65 pg/ml), regardless of the calcium level and hypocalcemia symptoms. Patients with a marked increase ≥1 pg/ml from the lowest post-operative PTH value were considered to have elevated serum PTH levels. Restoration was considered when the serum PTH level became normal.

### Statistical analysis

Univariate and multivariate analyses based on logistic regression and Cox regression were conducted. Normally distributed continuous data are presented as the mean ± standard deviation, and nonnormally distributed continuous data are presented as the median (interquartile range). Variables that were statistically significant or with a p value <0.10 in univariate analysis were enrolled in multivariate analysis to identify the independent factors. Logistic regression analyses are presented as odds ratios (ORs) and 95% confidence intervals (CIs), and Cox regression analyses are presented as hazard rates (HRs) and 95% CIs. SPSS (IBM SPSS Statistics 23.0, USA) and R software (Version 4.1.0, www.r-project.org, Vienna, Austria) were used for data analysis. Nomograms were formulated based on the results of multivariate Cox regression or logistic regression analyses by using the “rms” package in R. In all analyses, a two-sided p value < 0.05 was considered to be statistically significant.

### Ethical approval

The study was conducted in accordance with the principles and guidelines of the Declaration of Helsinki. The research protocol was approved by the Medical Ethics Committee of Chongqing General Hospital (KYS2022-005-01), and was granted informed consent exemption.

## Results

**Figure 1:**
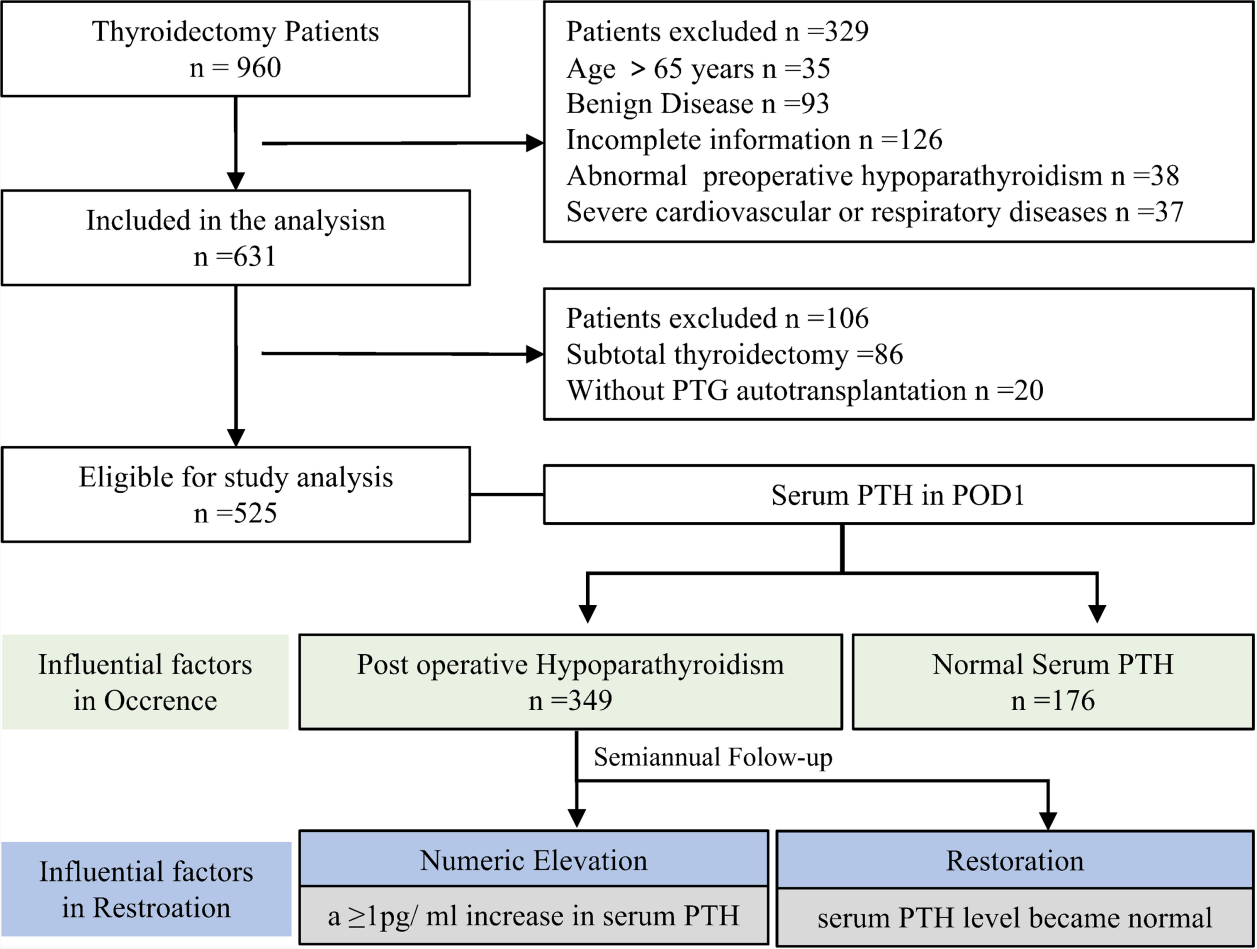
Study flowchart.

Data from 960 patients who underwent thyroidectomy from June 2020 to June 2021 in our hospital were collected. According to the inclusion and exclusion criteria, 525 patients with thyroid cancer who underwent TTIPA were finally included in the study. Among them, 349 patients (66.48%) had PTH levels lower than the normal value (12 pg/ml) on the first postoperative day and were diagnosed with POH. Logistic regression was used to explore the factors affecting the occurrence of postoperative POH in patients, and the half-year follow-up data of these patients were further collected. We aimed to explore the factors that affect the increase and restoration of PTH to normal after the operation. Thus, we defined a postoperative serum PTH level ≥1 pg/ml as an increase in the PTH value, and a PTH level that reached normal levels (≥12 pg/ml) was defined as the restoration of PTH. The time at which the value increased and the time it took to return to normal were recorded, and the influencing factors were explored through Cox regression (**Figure 1**). There were 90 males and 435 females in the study cohort, and the average age was 39.38 ± 10.89 years. Additionally, 84.76% of these patients underwent conventional conventional open thyroidectomy, and 15.24% of patients underwent endoscopic thyroidectomy. All surgical patients had at least one parathyroid gland transplanted (**Table 1**).

### Influencing factors of hypoparathyroidism after TTIPA

Taking the occurrence of postoperative POH as the outcome variable, the preoperative and intraoperative variables and the basic characteristics of the patients were all included as potential influencing factors for logistic univariate regression analysis. The factors with significant differences (P<0.05) were further analyzed by multivariate logistic regression analysis to explore the factors affecting POH after TTIPA in patients with thyroid cancer. The results showed that endoscopic thyroid surgery (OR=0.39, 95% CI: 0.22-0.68, P=0.001) was a protective factor for POH and that Hashimoto’s thyroiditis (OR=1.93, 95% CI: 1.09-3.42), P=0.024), the number of transplanted parathyroid glands (OR=2.70, 95% CI: 1.91-3.83, P<0.001) and blood glucose levels on the first postoperative day (OR=1.36, 95% CI: 1.06-1.74, P=0.016) were risk factors for POH. Preoperative PTH (OR = 0.99, 95% CI: 0.98-1.00, P=0.011) showed little influence on POH (**Table 2**).

**Table 2:** Infulential factors of POH in patients underwent TTIPA - Multivatirate and Univariate Logistic model.

Variables	Univariable Analysis		Multivariable Analysis	
	OR (95% CI)	*P*-value	OR (95% CI)	*P-*value
Method of Thyroidectomy (Endoscopic =1, Open =0)	**0.27 (0.16-0.44)**	**<0.001**	**0.39 (0.22-0.68)**	**0.001**
Hashimoto thyroiditis (Yes =1, No= 0)	**2.58 (1.58-4.20)**	**<0.001**	**1.93 (1.09-3.42)**	**0.024**
Serum glucose in POD1	**1.32 (1.08-1.62)**	**0.010**	**1.36 (1.06-1.74)**	**0.016**
Preoperative PTH	**0.99 (0.98-1.00)**	**0.010**	**0.99 (0.98-1.00)**	**0.011**
Number of PTG autotransplantation	**3.36 (2.54-4.44)**	**<0.001**	**2.70 (1.91-3.83)**	**<0.001**
Intraoperative blood loss	**1.01 (1.00-1.01)**	**<0.001**	1.00 (0.99-1.01)	0.980
Central lymph node dissection (Unilateral= 1, Bilateral= 0)	**0.39 (0.25-0.62)**	**<0.001**	0.93 (0.54-1.60)	0.793
Lateral lymph node dissection (Yes =1, No =0)	**1.01 (1.00-1.01)**	**<0.001**	1.00 (1.00-1.01)	0.095
PTG autotransplantation with same side CLND (Yes =1, No= 0)	**2.55 (1.50-4.32)**	**<0.001**	1.38 (0.73-2.61)	0.317
Age	1.00 (0.98-1.02)	0.980		
BMI	0.99 (0.94-1.04)	0.720		
Duration of operation	1.00(1.00-1.01)	0.130		
Preoperative serum glucose	0.95 (0.78-1.17)	0.660		
Preoperative serum calcium	1.65 (0.40-6.89)	0.490		
FNA (Yes =1, No = 0)	1.4 0 (0.96-2.05)	0.080		
Gender (Female =1, Male =0)	1.40 (0.88-2.24)	0.150		
BRAF V600E mutation status (Mutation =1, Wild type = 0)	1.23 (0.84-1.81)	0.290		
Lymph node metastisis, pathology (Yes =1, No =0)	1.07 (0.74-1.53)	0.730		

POH, Post-operative hypoparathyroidism; TTIPA, Total thyroidectomy with intraoperative parathyroid autotransplantation; POD number, Post-operative day number; PTH, Parathyroid hormone; PTG, Parathyroid gland; CLND, Central lymph node dissection; BMI, Body Mass Index; FNA, Fine needle aspiration.

### Factors influencing PTH increase after TTIPA

**Figure 2:**
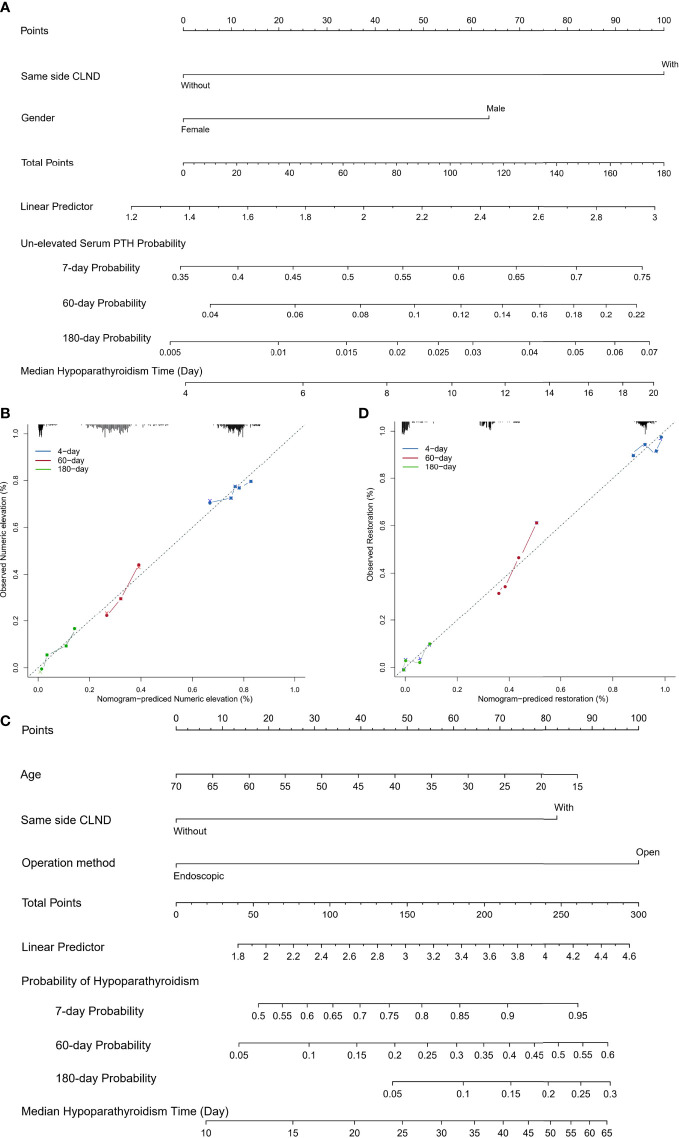
**(A)** Nomogram predicting the porbability of un-elevated serum PTH for TTIPA patients. Influential factors screened by multivariate Cox regression are listed along with related point axis. The sum of the scores for all variables is labeled on the total points axis, and 7-day, 60-day and 180-day porbabilities are drawn to determine the likelihood of un-elevated serum PTH; **(B)** Calibration curve of nomogram predicting the porbability of un-elevated serum PTH; **(C)** Nomogram predicting 7-day, 60-day and 180-day porbabilities of hypoparathyroidism. **(D)** Calibration curve of nomogram predicting the porbability of hypoparathyroidism.

Elevated PTH is an important sign of parathyroid function restoration. To explore the factors that affect the increase in PTH after TTIPA, we defined a ≥ 1 pg/ml increase in serum PTH levels as numerical elevation. Univariate Cox regression analyses were used to explore the influencing factors of PTH in the early stage, and factors with significant differences (P < 0.05) were included in multivariate Cox regression to reduce the effect of the interaction. The results showed that PTG autotransplantation patients with same side CLND (HR=0.50; 95% CI: 0.34-0.73, P<0.001) had a longer time to PTH increase after transplantation, and female patients (HR=1.35, 95% CI: 1.00-1.81, P=0.047) were more prone to PTH increases (**Table 3**). Furthermore, Cox regression was used to draw a nomogram. A prediction model was built for the increase in PTH values at 7 days, 60 days, and 180 days, and a calibration curve was used for calibration (**Figures 2A**, **B**).

**Table 3:** Infulential factors of PTH numeric elevation in patients underwent TTIPA - Multivatirate and Univariate Cox model.

Variables	Univariable Analysis		Multivariable Analysis	
	HR (95% CI)	P-value	HR (95% CI)	P-value
PTG autotransplantation with same side CLND (Yes =1, No= 0)	**0.48 (0.33-0.70)**	**<0.001**	**0.50 (0.34-0.73)**	**<0.001**
Gender (Female =1, Male =0)	**1.40 (1.04-1.87)**	**0.026**	**1.35 (1.00-1.81)**	**0.047**
Age	1.00 (0.99-1.01)	0.411		
BMI	0.99 (0.96-1.02)	0.548		
Duration of operation	1.00 (1.00-1.00)	0.606		
Post-operative drainag	1.00 (1.00-1.00)	0.834		
Serum glucose in POD1	1.00 (0.93-1.07)	0.947		
Intraoperative blood loss	1.00 (1.00-1.00)	0.092		
Preoperative serum glucose	1.10 (1.00-1.20)	0.051		
Average hospitalization time	1.00 (0.94-1.07)	0.892		
Number of PTG autotransplantation	0.97 (0.84-1.12)	0.672		
FNA (Yes =1, No = 0)	1.03 (0.83-1.27)	0.797		
Method of thyroidectomy (Endoscopic =1, Open =0)	1.22 (0.85-1.75)	0.289		
Hashimoto thyroiditis (Yes =1, No= 0)	1.19 (0.94-1.50)	0.145		
BRAF V600E mutation status (Mutation =1, Wild type = 0)	1.01 (0.90-1.14)	0.819		
Central lymph node dissection (Unilateral= 1, Bilateral= 0)	1.09 (0.95-1.24)	0.218		
Lateral lymph node dissection (Yes =1, No =0)	1.00 (1.00-1.00)	0.121		
Lymph node metastisis, pathology (Yes =1, No =0)	1.08 (0.87-1.33)	0.496		

PTH, Parathyroid hormone; TTIPA, Total thyroidectomy with intraoperative parathyroid autotransplantation; PTG, Parathyroid gland; CLND, Central lymph node dissection; BMI, Body Mass Index; POD number, Post-operative day number; FNA, Fine needle aspiration.

### Factors affecting restoration of PTH to the reference range after operation

Furthermore, in order to clarify the factors that affect the restoration of PTH in patients, univariate COX regression analyses were used to find that age (from 18 to 70-year-old, HR=1.01, 95% CI: 1.00-1.02, P=0.049), PTG autotransplantation with/without same side CLND (with vs. without, HR=0.56, 95% CI: 0.38-0.82, P=0.003) and endoscopic surgery (endoscopic vs. open, HR=1.57, 95% CI: 1.06-2.32, P=0.023) were influential factors for the restoration of PTH to the reference range within six months. The multivariate COX regression indicated that PTG autotransplantation with same side CLND (HR=0.56, 95% CI: 0.38-0.82, P=0.003) patients had a longer time to PTH restoration, and patients with endoscopic surgery (HR=1.54, 95% CI: 1.04-2.28, P=0.029) were more likely to recover within six months. These findings suggest that CLND and open surgery on the same side of the transplant may be key factors affecting the restoration of TTIPA patients to normal (**Table 4**). Furthermore, based on Cox regression, a nomogram prediction model for 7-day, 60-day, and 180-day PTH restoration was constructed and calibrated using the calibration curve (**Figures 2C**, **D**).

**Table 4:** Infulential factors of PTH restoration in patients underwent TTIPA - Multivatirate and Univariate Cox model.

Variables	Univariable Analysis		Multivariable Analysis	
	HR (95% CI)	*P*-value	HR (95% CI)	*P*-value
Age	**1.01 (1.00-1.02)**	**0.049**	1.01 (1.00-1.02)	0.056
PTG autotransplantation with same side CLND (Yes =1, No= 0)	**0.56 (0.38-0.82)**	**0.003**	**0.56 (0.38-0.82)**	**0.003**
Method of thyroidectom (Endoscopic =1, Open =0)	**1.57 (1.06-2.32)**	**0.023**	**1.54 (1.04-2.28)**	**0.029**
BMI	1.00 (0.97-1.03)	0.910		
Duration of operation	1.00 (1.00-1.00)	0.640		
Serum glucose in POD1	1.00 (0.93-1.09)	0.932		
Intraoperative blood loss	1.00 (1.00-1.00)	0.600		
Post-operative drainag	1.00 (1.00-1.00)	0.920		
Preoperative serum glucose	1.01 (0.89-1.15)	0.882		
Average hospitalization time	0.99 (0.93-1.06)	0.807		
Number of PTG autotransplantation	0.92 (0.79-1.06)	0.233		
FNA (Yes =1, No = 0)	0.98 (0.79-1.22)	0.887		
Gender (Female =1, Male =0)	1.21 (0.90-1.63)	0.203		
Hashimoto thyroiditis (Yes =1, No= 0)	1.05 (0.83-1.32)	0.692		
BRAF V600E mutation status (Mutation =1, Wild type = 0)	0.99 (0.88-1.12)	0.895		
Lateral lymph node dissection (Yes =1, No =0)	1.00 (1.00-1.00)	0.854		
Central lymph node dissection (Unilateral= 1, Bilateral= 0)	1.09 (0.95-1.24)	0.209		
Lymph node metastisis, pathology (Yes =1, No =0)	1.00 (0.81-1.24)	0.963		

PTH, Parathyroid hormone; TTIPA, Total thyroidectomy with intraoperative parathyroid autotransplantation; PTG, Parathyroid gland; CLND, Central lymph node dissection; BMI, Body Mass Index; POD number, Post-operative day number; FNA, Fine needle aspiration.

## Discussion

Thyroid cancer is the most common cervical malignancy, and total thyroidectomy is the main treatment for thyroid cancer. Due to the complex structure of the neck, patients often suffer from various complications, such as hemorrhage, infection, and nerve damage, after surgery (3). Among them, 30-60% of patients may develop POH (4). Hypoparathyroidism is a syndrome of hypocalcemia and hyperphosphatemia caused by decreased PTH. It is mainly characterized by increased excitability of the neuromuscular system with abnormal systemic calcium and phosphorus metabolism, such as multitissue metastatic calcification and ectodermal tissue degeneration (13). Several previous studies and our long-term clinical practice have found that most patients have transient hypoparathyroidism; that is, plasma PTH levels can be recovered within six months. Furthermore, approximately 0.5-15% of patients have long-term hypoparathyroidism (14, 15). Therefore, it is of great importance to explore the risk factors for the occurrence of POH and to clarify the influencing factors of postoperative PTH restoration in patients with POH.

Intraoperative parathyroid transplantation is the main method to maintain the physiological level of PTH in patients (16). Most studies believe that parathyroid transplantation during total thyroidectomy can prevent POH to a certain extent and reduce the occurrence of permanent hypoparathyroidism (17, 18). However, Matilda Annebäck et al. also pointed out that parathyroid transplantation is a risk factor for permanent hypoparathyroidism in patients (OR: 1.72; 95% CI: 1.47-2.01), suggesting that surgical methods, surgeons and transplantation strategies may have an important influence on the restoration of PTH in patients (11). To further clarify the influencing factors of postoperative POH and PTH restoration in patients undergoing TTIPA, we reviewed the data of thyroid cancer patients who underwent TTIPA by the same experienced physicians from June 2020 to June 2021 in our department. Our study found that the proportion of POH in TTIPA patients was approximately 37%, and 37% of these patients recovered to physiological levels before discharge. Furthermore, some patients recovered within 2 months, and approximately 1.90% of patients did not recover to physiological levels within six months.

A number of previous studies have indicated that the preoperative PTH level and the number of transplanted PTGs are related factors affecting the occurrence of POH (16, 19). Similarly, our multivariate analysis also showed that the preoperative PTH level and the number of transplanted PTGs were influencing factors of POH in TTIPA patients. In addition, the postoperative fasting blood glucose level, surgical method (open vs. endoscopic) and Hashimoto’s thyroiditis were also factors affecting the incidence of POH in TTIPA patients. Although most studies suggest that there is no significant difference in the incidence of POH between endoscopic surgery and open surgery, some studies have indicated that the incidence of permanent hypoparathyroidism in endoscopic surgery is lower than that in open surgery (20, 21). We focused on TTIPA patients and found that endoscopic surgery was a protective factor for POH compared with open surgery (OR=0.39, 95% CI: 0.22-0.68, P=0.001). One possible reason is that the exposure of the operative field of laparoscopy is more sufficient, which is helpful for identifying the parathyroid glands during the operation. The other possible reason is that the traction on the sternocleidomastoid muscle during laparoscopy is small, which may help protect the blood supply and function of the sternocleidomastoid muscle and thus promote graft survival (22, 23).

There are few studies clarifying the effect of blood glucose on PTH in patients with TTIPA. Marie Reeberg Sass et al. analyzed parathyroid surgery specimens. They found that serum PTH levels were correlated with serum insulin levels and that parathyroid cells expressed insulin receptors (24). Ruizhi Jiajue et al. indicated that PTH levels in patients with diabetes were lower than normal (25). The above studies suggest that there may be a complex regulatory relationship between blood glucose levels, insulin levels and postoperative PTH levels. Future studies should explore the role of perioperative blood glucose monitoring and insulin use in maintaining postoperative PTH levels and reducing the risk of POH.

In addition, previous studies have demonstrated that thyroid surgery with Hashimoto’s thyroiditis (HT) has a significantly increased risk of postoperative complications (26). This is similar to our results, approximately 23.8% of thyroid cancer patients had Hashimoto’s thyroiditis. The logistic multivariate analysis showed that Hashimoto’s thyroiditis was an independent risk factor for POH (OR=1.93, 95% CI: 1.09-3.42, P=0.024). One possible reason is that the reactive enlarged lymph nodes in patients with HT combined with PTC may be misdiagnosed as metastatic lymph nodes. Thus, the probability of lymph node dissection is higher, the damage to the operation area is worse, and the parathyroid gland damage increases (27). Another possible reason is that bridge changes in thyroid mass (fragility, vascularity (rich blood supply), and/or fibrosis) in thyroiditis can significantly prolong operative time and increase operative difficulty (28). Thus, it can increase the probability of parathyroid injury and the blood supply of the sternocleidomastoid muscle in the transplant area.

We further evaluated the factors influencing the PTH increases and PTH restoration to normal. Although approximately 98% of patients eventually returned to the reference range, there were differences in the speed of restoration. In POH patients, a ≥ 1 pg/ml increase in PTH levels was used as the end point event. The factors affecting the elevated level of PTH were explored, and the corresponding time was recorded. Univariate and multivariate Cox regression indicated that female patients or the ipsilateral transplant did not undergo central surgery. Patients with regional lymph node dissection (PTG autotransplantation with same-side CLND (HR=0.50; 95% CI: 0.34-0.73, P<0.001) had an earlier rise in PTH levels. The role of sex in the restoration of PTH in patients after PTG transplantation has not been reported, but multiple studies suggest that female patients are at a higher risk of developing postthyroidectomy hypoparathyroidism for TTIPA patients (29).

Although we did not find a statistical relationship between the incidence of POH and sex, it seems that female patients do have a higher risk (female vs. male, HR=1.40, 95% CI=0.88-2.24). This finding suggests that PTH levels may be regulated by estrogen. More importantly, estrogen can directly or indirectly promote the expression of PTH-related genes and PTH secretion (30, 31). Almaden, Y. et al. further confirmed that female parathyroid tissue had a stronger proliferation ability (32). More importantly, studies suggest that women tend to benefit more than men during transplantation of various tissues, including islets and lymph nodes. These findings suggest that estrogen may play a key role in promoting graft survival and growth (33). However, as e number of female patients underwent TTIPA was remarkably higher than male patients, a unavoidable sample size bias may introduce this statistical differences. Therefore, future clinical studies with larger samples are needed to explain the role of gender in promoting the restoration of POH. Additionally, exploring the role of sex and estrogen in the regulation of PTH and its influence on PTH restoration in patients with TTIPA may be an important direction to shorten the restoration time of PTH and reduce the occurrence of POH.

In addition, we found that time to PTH elevation and restoration in PTG autotransplantated patients with same-side CLND was longer than those without CLND. The sternocleidomastoid muscle is the most commonly transplanted site of the parathyroid glands (34). Unfortunately, limited studies have analyzed the correlation between the sternocleidomastoid muscle transplantation location and the level of PTH. The blood supply of the sternocleidomastoid muscle mainly comes from the thyroid neck trunk, the superior thyroid artery and the occipital artery (35). During the dissection of the central lymph nodes, there may be damage to small blood vessels branching to the transverse carotid artery and the superior thyroid artery of the thyroid neck trunk (35). Excessive lateral sternocleidomastoid traction causes local muscle damage and affects the transplantation effect (36). Therefore, to ensure the restoration of PTH, it is of great importance to clarify the site of parathyroid transplantation. In addition to the sternocleidomastoid muscle, parathyroid gland transplantation can also be transplanted into the pectoral muscle, forearm muscle group, subcutaneous tissue, inguinal area, and abdominal cavity (34, 37). Further exploration of specific transplantation sites and transplantation strategies are important directions in the development of thyroid surgery under the guidance of precision medicine in the future. In addition to performing CLND, we also found that the surgical approach is an important factor affecting the restoration of PTH to reference range. Compared with patients undergoing open surgery, the time required for PTH to return to normal in patients with TTIPA undergoing endoscopic surgery is shorter. Combined with results listed above, this study revealed that the incidence of POH in patients with TTIPA undergoing open surgery is higher, and special attention should be given to parathyroid gland recognition and protection in patients who are planning to undergo open surgery.

## Conclusion

A high postoperative fasting blood glucose level, a large number of transplanted PTGs, open surgery and Hashimoto’s thyroiditis are risk factors for postoperative POH in TTIPA patients. Elevated PTH levels occur earlier in female patients and patients without CLND on the transplant side. PTH recovers to reference range earlier in patients without CLND and endoscopic surgery on the transplant side.

## Data availability statement

The raw data supporting the conclusions of this article will be made available by the authors, without undue reservation.

## Ethics statement

The studies involving human participants were reviewed and approved by Medical Ethics Committee of Chongqing General Hospital. The patients/participants provided their written informed consent to participate in this study. Written informed consent was obtained from the individual(s) for the publication of any potentially identifiable images or data included in this article.

## Author contributions

DL and ET contributed to the conceptualization of the study, data collection and interpretation, and drafting of the manuscript. ZYa and DL. contributed to analyzed the data. SY, JZ, HM contributed to data collection and interpretation, ZYi, FC, YS, YL, TY reviewed and edited the manuscript. FZ and ZYa contributed to the data interpretation, project administration. All authors reviewed the article and approved submission.

## Funding

This work was supported by the Chongqing Medical Scientific Research Project (Joint Project of Chongqing Health Commission and Science and Technology Bureau) (Grant No. 2021MSXM314) and the Medical Science and Technology Innovation Fund of Chongqing General Hospital (Grant No. 2019ZDXM01).

## Conflict of interest

The authors declare that the research was conducted in the absence of any commercial or financial relationships that could be construed as a potential conflict of interest.

## Publisher’s note

All claims expressed in this article are solely those of the authors and do not necessarily represent those of their affiliated organizations, or those of the publisher, the editors and the reviewers. Any product that may be evaluated in this article, or claim that may be made by its manufacturer, is not guaranteed or endorsed by the publisher.

## References

[1] Miranda-Filho A Lortet-Tieulent J Bray F Cao B Franceschi S Vaccarella S. Thyroid cancer incidence trends by histology in 25 countries: a population-based study. Lancet Diabetes Endocrinol (2021) 9(4):225–34. doi: 10.1016/S2213-8587(21)00027-9 33662333

[2] Nabhan F Dedhia PH Ringel MD. Thyroid cancer, recent advances in diagnosis and therapy. Int J Cancer (2021) 149(5):984–92. doi: 10.1002/ijc.33690 34013533

[3] Kazaure HS Sosa JA. Surgical hypoparathyroidism. Endocrinol Metab Clin North Am (2018) 47(4):783–96. doi: 10.1016/j.ecl.2018.07.005 30390813

[4] Edafe O Antakia R Laskar N Uttley L Balasubramanian SP. Balasubramanian SP. systematic review and meta-analysis of predictors of post-thyroidectomy hypocalcaemia. Br J Surg (2014) 101(4):307–20. doi: 10.1002/bjs.9384 24402815

[5] Koimtzis GD Stefanopoulos L Giannoulis K Papavramidis TS. What are the real rates of temporary hypoparathyroidism following thyroidectomy? it is a matter of definition: a systematic review. Endocrine (2021) 73(1):1–7. doi: 10.1007/s12020-021-02663-8 33651345

[6] Testini M Gurrado A Lissidini G Nacchiero M. Hypoparathyroidism after total thyroidectomy. Minerva CHIR (2007) 62(5):409–15. https://europepmc.org/article/MED/17947951 17947951

[7] Ponce DLG Bonilla-Ramirez C Hernandez-Calderon FJ Pantoja-Millan JP Sierra-Salazar M Velazquez-Fernandez D. Mid-term and long-term impact of permanent hypoparathyroidism after total thyroidectomy. World J Surg (2020) 44(8):2692–8. doi: 10.1007/s00268-020-05531-0 32322939

[8] Qiu Y Xing Z Qian Y Fei Y Luo Y Su A. Selective parathyroid autotransplantation during total thyroidectomy for papillary thyroid carcinoma: A cohort study. Front Surg (2021) 8:683041. doi: 10.3389/fsurg.2021.683041 34262932 PMC8274712

[9] Godlewska P Benke M Stachlewska-Nasfeter E Galczynski J Pula B Dedecjus M. Risk factors of permanent hypoparathyroidism after total thyroidectomy and central neck dissection for papillary thyroid cancer: a prospective study. Endokrynol Pol (2020) 71(2):126–33. doi: 10.5603/EP.a2020.0006 32154569

[10] Eismontas V Slepavicius A Janusonis V Zeromskas P Beisa V Strupas K. Predictors of postoperative hypocalcemia occurring after a total thyroidectomy: results of prospective multicenter study. BMC Surg (2018) 18(1):55. doi: 10.1186/s12893-018-0387-2 30092793 PMC6085643

[11] Anneback M Hedberg J Almquist M Stalberg P Norlen O. Risk of permanent hypoparathyroidism after total thyroidectomy for benign disease: A nationwide population-based cohort study from Sweden. Ann Surg (2021) 274(6):e1202–8. doi: 10.1097/SLA.0000000000003800 32032086

[12] Yin S Pan B Yang Z Tang M Mo H Li Y. Combined use of autofluorescence and indocyanine green fluorescence imaging in the identification and evaluation of parathyroid glands during total thyroidectomy: A randomized controlled trial. Front Endocrinol (2022) 13. doi: 10.3389/fendo.2022.897797 PMC9243533 35784544

[13] Hans SK Levine SN. Hypoparathyroidism. (2022). In: StatPearls. Treasure Island (FL): StatPearls Publishing; May 15, 2022

[14] Qiu Y Xing Z Fei Y Qian Y Luo Y Su A. Role of the 2018 American thyroid association statement on postoperative hypoparathyroidism: a 5-year retrospective study. BMC Surg (2021) 21(1):334. doi: 10.1186/s12893-021-01333-w 34474672 PMC8414735

[15] Stack BJ Bimston DN Bodenner DL Brett EM Dralle H Orloff LA. American Association of clinical endocrinologists and American college of endocrinology disease state clinical review: Postoperative hypoparathyroidism–definitions and management. Endocr Pract (2015) 21(6):674–85. doi: 10.4158/EP14462.DSC 26135962

[16] Qiu Y Fei Y Xing Z Zhu J Luo Y Su A. Does the number of autotransplanted parathyroid glands affect postoperative hypoparathyroidism and serum parathyroid hormone levels? Asian J Surg (2022) 45(1):117–24. doi: 10.1016/j.asjsur.2021.03.031 33863630

[17] Wang B Zhu CR Yao XM Wu J. The effect of parathyroid gland autotransplantation on hypoparathyroidism after thyroid surgery for papillary thyroid carcinoma. Cancer Manag Res (2021) 13:6641–50. doi: 10.2147/CMAR.S323742 PMC8402957 34466034

[18] Wang B Zhu CR Liu H Wu J. The effectiveness of parathyroid gland autotransplantation in preserving parathyroid function during thyroid surgery for thyroid neoplasms: A meta-analysis. PloS One (2019) 14(8):e221173. doi: 10.1371/journal.pone.0221173 PMC6693848 31412080

[19] Huang R Wang Q Zhang W Zha S Jiang D Xu X. The predictive factors for postoperative hypoparathyroidism and its severity on the first postoperative day after papillary thyroid carcinoma surgery. Eur Arch Otorhinolaryngol (2021) 278(4):1189–98. doi: 10.1007/s00405-020-06211-4 32691233

[20] Sun H Wang X Zheng G Wu G Zeng Q Zheng H. Comparison between transoral endoscopic thyroidectomy vestibular approach (TOETVA) and conventional open thyroidectomy for patients undergoing total thyroidectomy and central neck dissection: A propensity score-matching analysis. Front Oncol (2022) 12:856021. doi: 10.3389/fonc.2022.856021 35311081 PMC8925319

[21] Akritidou E Douridas G Spartalis E Tsourouflis G Dimitroulis D Nikiteas NI. Complications of trans-oral endoscopic thyroidectomy vestibular approach: A systematic review. In Vivo (2022) 36(1):1–12. doi: 10.21873/invivo.12671 34972695 PMC8765134

[22] Zhang H Shi W Zhang J Xu J Zhou D Liu W. Comparing endoscopic thyroidectomy using the breast approach and conventional open thyroidectomy: A retrospective analysis. J Cancer Res Ther (2021) 17(5):1248–52. doi: 10.4103/jcrt.jcrt_707_21 34850774

[23] Lin P Han P Liang F Cai Q Chen R Yu S. Characteristics of the parathyroid gland in endoscopic thyroidectomy with the application of an image enhancement system. Surg Endosc (2018) 32(9):3925–35. doi: 10.1007/s00464-018-6132-1 29488092

[24] Sass MR Wewer AN Pedersen J Hare KJ Borbye-Lorenzen N Kiss K. Secretion of parathyroid hormone may be coupled to insulin secretion in humans. Endocr Connect (2020) 9(7):747–54. doi: 10.1530/EC-20-0092 PMC7424341 32698134

[25] Jiajue R Liu S Pei Y Qi X Jiang Y Wang Q. Associations between osteocalcin, calciotropic hormones, and energy metabolism in a cohort of Chinese postmenopausal women: Peking vertebral fracture study. Int J Endocrinol (2021) 2021:5585018. doi: 10.1155/2021/5585018 33833796 PMC8016567

[26] McManus C Luo J Sippel R Chen H. Is thyroidectomy in patients with hashimoto thyroiditis more risky? J Surg Res (2012) 178(2):529–32. doi: 10.1016/j.jss.2012.09.017 PMC3496049 23043868

[27] Donangelo I Walts AE Bresee C Braunstein GD. Lymphocytic thyroiditis is associated with increased number of benign cervical nodes and fewer central neck compartment metastatic lymph nodes in patients with differentiated thyroid cancer. Endocr Pract (2016) 22(10):1192–8. doi: 10.4158/E151078.OR 27732096

[28] Saadi R Brandt A Kim Y Cottrill E Saunders B Schaefer E. Degree of technical difficulty of thyroidectomy for autoimmune thyroid disease. Head Neck (2020) 42(2):262–8. doi: 10.1002/hed.25991 31651072

[29] Sands NB Payne RJ Cote V Hier MP Black MJ Tamilia M. Female gender as a risk factor for transient post-thyroidectomy hypocalcemia. Otolaryngol Head Neck Surg (2011) 145(4):561–4. doi: 10.1177/0194599811414511 21750342

[30] Naveh-Many T Almogi G Livni N Silver J. Estrogen receptors and biologic response in rat parathyroid tissue and c cells. J Clin Invest (1992) 90(6):2434–8. doi: 10.1172/JCI116134 PMC443399 1469095

[31] Naveh-Many T Epstein E Silver J. Oestrogens and calcium regulatory hormones: potential implications for bone. Curr Opin Nephrol Hypertens (1995) 4(4):319–23. doi: 10.1097/00041552-199507000-00006 7552097

[32] Almaden Y Felsenfeld AJ Rodriguez M Canadillas S Luque F Bas A. Proliferation in hyperplastic human and normal rat parathyroid glands: role of phosphate, calcitriol, and gender. Kidney Int (2003) 64(6):2311–7. doi: 10.1046/j.1523-1755.2003.00331.x 14633156

[33] Lemos J Baidal DA Poggioli R Fuenmayor V Chavez C Alvarez A. Prolonged islet allograft function is associated with female sex in patients after islet transplantation. J Clin Endocrinol Metab (2022) 107(3):e973–9. doi: 10.1210/clinem/dgab787 PMC8852206 34727179

[34] Iorio O Petrozza V De Gori A Bononi M Porta N De Toma G. Parathyroid autotransplantation during thyroid surgery. where we are? a systematic review on indications and results. J Invest Surg (2019) 32(7):594–601. doi; 10.1080/08941939.2018.1441344 29658811

[35] Kierner AC Aigner M Zelenka I Riedl G Burian M. The blood supply of the sternocleidomastoid muscle and its clinical implications. Arch Surg (1999) 134(2):144–7. doi: 10.1001/archsurg.134.2.144 10025452

[36] Rodriguez-Torres J Lopez-Lopez L Cabrera-Martos I Torres-Sanchez I Ortiz-Rubio A Valenza MC. Musculoskeletal neck disorders in thyroid cancer patients after thyroidectomy. Eur J Cancer Care (Engl) (2019) 28(4):e13053. doi: 10.1111/ecc.13053 31016824

[37] Cui Q Kong D Li Z Wang K Zhang D Tang J. Parathyroid autotransplantation at a novel site for better evaluation of the grafted gland function: study protocol for a prospective, randomized controlled trial. Trials (2019) 20(1):96. doi: 10.1186/s13063-019-3195-9 30704522 PMC6357396

